# Instrumental variable analysis to estimate treatment effects: a simulation study showing potential benefits of conditioning on hospital

**DOI:** 10.1186/s12874-022-01598-6

**Published:** 2022-04-25

**Authors:** I. E. Ceyisakar, N. van Leeuwen, E. W. Steyerberg, H. F. Lingsma

**Affiliations:** 1 Centre for Medical Decision Making, Department of Public Health, Erasmus MC – University Medical Center Rotterdam, PO Box 2040, 3000 CA Rotterdam, The Netherlands; 2 Department of Biomedical Data Sciences, Leiden University Medical Centre, Leiden, the Netherlands

**Keywords:** Confounding by indication, Unmeasured confounders, Instrumental variable analysis, Between-hospital variation, Observational data, Comparative effectiveness research

## Abstract

**Background:**

Instrumental variable (IV) analysis holds the potential to estimate treatment effects from observational data. IV analysis potentially circumvents unmeasured confounding but makes a number of assumptions, such as that the IV shares no common cause with the outcome. When using treatment preference as an instrument, a common cause, such as a preference regarding related treatments, may exist. We aimed to explore the validity and precision of a variant of IV analysis where we additionally adjust for the provider: adjusted IV analysis.

**Methods:**

A treatment effect on an ordinal outcome was simulated (beta − 0.5 in logistic regression) for 15.000 patients, based on a large data set (the IMPACT data, *n* = 8799) using different scenarios including measured and unmeasured confounders, and a common cause of IV and outcome. We compared estimated treatment effects with patient-level adjustment for confounders, IV with treatment preference as the instrument, and adjusted IV, with hospital added as a fixed effect in the regression models.

**Results:**

The use of patient-level adjustment resulted in biased estimates for all the analyses that included unmeasured confounders, IV analysis was less confounded, but also less reliable. With correlation between treatment preference and hospital characteristics (a common cause) estimates were skewed for regular IV analysis, but not for adjusted IV analysis.

**Conclusion:**

When using IV analysis for comparing hospitals, some limitations of regular IV analysis can be overcome by adjusting for a common cause.

**Trial registration:**

We do not report the results of a health care intervention.

**Supplementary Information:**

The online version contains supplementary material available at 10.1186/s12874-022-01598-6.

## Background

The use of observational data to assess the effectiveness of treatments [1] is a useful alternative when randomized controlled trials (RCTs) are not feasible. The most important methodological challenge in observational data is to estimate the causal relation between the treatment and outcome while avoiding confounding by indication [2, 3]. The method most commonly reported to avoid confounding by indication in observational data, is controlling for known prognostic factors [4] using regression analysis. Another way to account for the differences in baseline characteristics between treated and non-treated patients in observational data is propensity scores. These methods share the caveat that any unmeasured confounders could still bias the results [5, 6].

Instrumental variable (IV) methods provide a possibility to estimate the effects of treatment in the presence of unmeasured confounders, circumventing confounding by indication [7]. Treatment preference of a provider is one of the popular instrumental variables used [8].

For many medical treatments, such as treatment for traumatic brain injury, effectiveness has not been fully determined [9], leading to a wide variation in treatment policies being applied across different hospitals [10]. Local preferences may cause interventions to become standard practice in some hospitals, but not in others. This provides the possibility to use IV methods with treatment preference as an instrumental variable to evaluate effectiveness of commonly applied treatments in observational data.

**Fig. 1:**
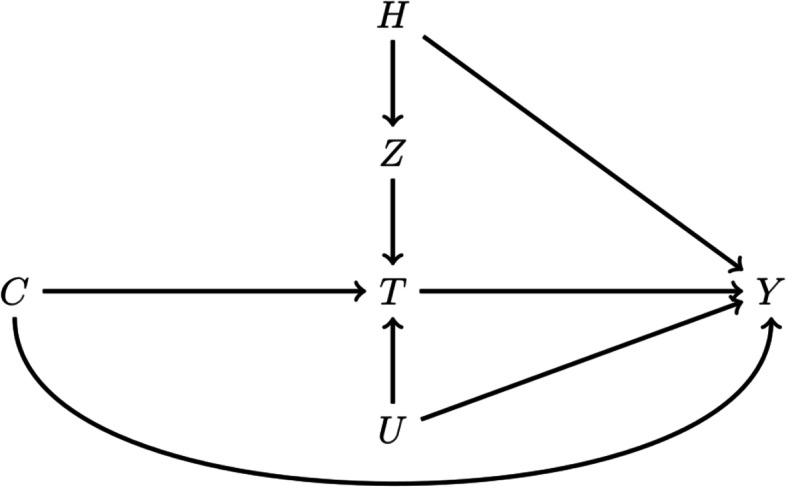
A directed acyclic graph (DAG) showing the causal assumption of the observational data and confounding caused by alternative pathways through the unobserved (U) confounders and through hospital (H). H: hospital. Z: treatment preference as instrument: proportion of treated patients within each hospital. T: treatment. C: patient characteristics. PS: propensity score. Y: outcome. U: unobserved confounders

IV analyses has three main assumptions; (*relevance*) that there is an instrument and that this instrument is associated with the exposure, (*exclusion restriction*) the IV can only affect the outcome through treatment, and (*exchangeability*) the outcome and the IV cannot share a common cause [11]. The first assumption can be empirically verified, while the second and third can’t be tested. All three assumptions will be addressed in this paper with the main concern being *exchangeability,* since hospitals might differ systemically in some general characteristics which might be associated with the treatment preference of interest. We therefore assume correlation between hospital characteristics such as quality of care and treatment preference exist (Fig. 1). To address this assumption we adjusted for hospital, which in theory would capture differences between hospitals that affect outcome other than treatment preference [12, 13].

Our aim is to assess the validity and precision of *adjusted IV* analysis compared to regular IV analysis, adjustment at patient level and propensity score matching to estimate treatment effectiveness in observational data in the context of between-hospital variation. We hereto perform a simulation study motivated by a comparative effectiveness study in traumatic brain injury.

## Methods

### Motivating data

Simulations were based on a dataset with traumatic brain injury patients. The traumatic brain injury field is known to have a large between hospital variation [14]. The International Mission for Prognosis and Analysis of Clinical Trials (IMPACT) dataset used, is a combination of data from prospective studies and phase III trials in patients with moderate and severe traumatic brain injury, and has been described in detail in previous studies [15–17]. The treatment used for comparison was insertion of an intracranial pressure monitor since this is an intervention of which effectiveness is currently still uncertain [18]. Patients with severe traumatic brain injury either receive this intervention or not, depending on the prognostic factors. The following prognostic factors were sampled with replacement from the IMPACT database and used for the analyses: Glasgow Coma Scale motor score, age, sex, pupillary reactivity, the presence of subarachnoid hemorrhages and the Traumatic Coma Data Bank computed tomography scan classification (henceforth CT scan classification) [19, 20].

### Simulated data

We simulated a hypothetical dataset of patients, receiving treatment and not receiving treatment, distributed over different hospitals (H) in five steps; 1) Patients get a value for the patient characteristics, 2) Treatment preference of each hospital is randomly assigned, which gives patients a higher or lower chance on receiving treatment depending on which center they are treated 3) Overall performance of the hospital (H) is randomly assigned, which gives patients a better or worse outcome depending on which center they were treated, partly independent of the treatment preference, 4) whether a patient is treated (T) is predicted using patient characteristics (C, U) and preference of the hospitals (H_p_) in the model *T* = *C* ∗ *β*_*CT*_ + *U* ∗ *β*_*UT*_ + *H*_*p*_ ∗ *β*_*hT*_ and 5) the outcome Y (four level ordinal scale) per patient is generated using patient characteristics (C, U), the treatment (T) and overall hospital performance (H) in the model *Y* = *C* ∗ *β*_*CY*_ + *U* ∗ *β*_*UY*_ + *T* ∗ *β*_*T*_ + *H* ∗ *β*_*HY*_.

The patients (15000) for the simulation were sampled from the original IMPACT database (*n* = 8799), and were randomly assigned to hospitals (*N* = 100). Each patient had data on the above mentioned prognostic factors: GCS motor score, age, sex, pupillary reactivity, the presence of subarachnoid hemorrhages and the CT scan classification [19]. The hypothetical dichotomous treatment variable was simulated with a beneficial treatment effect (OR = 1.65, corresponding to a Beta of − 0.5) on outcome. To include hospital treatment preference every hospital in the simulation received a random percentage within the range of variation of intracranial pressure monitoring among hospitals observed in empirical data (17 to 58%) [21]. Proportional odds ordinal regression models were used to estimate the impact of each of the prognostics factors on the outcome and binary logistic regression models on the decision to assign treatment [21]. The outcome generated was the Glasgow Outcome Scale (GOSE) at 6 months, collapsed into a four-point ordinal scale (death and vegetative state were combined for ethical reasons).

The treatment preference and the center effect were correlated (mean correlation coefficient 0.3). We repeated the simulation with higher correlations (mean correlation coefficient 0.5, mean correlation coefficient 0.8).

### Statistical analysis

Treatment effects were estimated with five different strategies (Table 1). In all analyses proportional odds ordinal logistic regression models were used. The prognostic factors pupillary reactivity, presence of subarachnoid hemorrhages and the CT scan classification were disregarded in all analysis to mimic unmeasured confounders (Table 2). In analysis (a) there was no adjustment for the observed confounders, this adjustment was added in analysis (b). In the propensity score adjusted model (c) the propensity score was estimated based on the observed confounders. The outcome was regressed on the estimated propensity score using again a proportional odds ordinal logistic regression model. In the first IV analysis (d) the same model was used as in (a) instead of the actual treatment which the patient would receive, the treatment preference (percentage of treated patients in a certain hospital) was added as a dependent variable. One extra control model was included in the last scenario, where all confounders (also unmeasured) as well as the IV were regressed on the outcome (Table 1).

**Table 1:** 5 different methods of analysis to estimate the association between treatment and outcome

	Analysis strategy	Formula
a	Univariable regression analysis	*logit*(*P*[*Y* + 1]) = *β*_0_ + *T* ∗ *β*_*T*_
b	Regression analysis with covariate adjustment	*logit*(*P*[*Y* + 1]) = *C* ∗ *β*_*C*_ + *T* ∗ *β*_*T*_
c	Propensity score adjustment	*PS* = *C* ∗ *β*_*C*_ *logit*(*P*[*Y* + 1]) = *PS* ∗ *β*_*PS*_ + *T* ∗ *β*_*T*_
d	IV analysis	*logit*(*P*[*Y* + 1]) = *Z* ∗ *β*_*Z*_
e	IV analysis with correction for hospital	*logit*(*P*[*Y* + 1]) = *Z* ∗ *β*_*Z*_ + *H* ∗ *β*_*H*_
f	IV analysis with correction for hospital and all measured and unmeasured confounders	*logit*(*P*[*Y* + 1]) = *Z* ∗ *β*_*Z*_ + *H* ∗ *β*_*H*_ + *C* ∗ *β*_*C*_ + *U* ∗ *β*_*U*_

Z: treatment preference as instrument: proportion of treated patients within each hospital

H: hospital

C: patient characteristics

PS: propensity score

T: treatment

β0: intercept

**Table 2:** Overview of the variables used as observed and unobserved confounders

**Observed confounders**	GCS motor score Age Sex
**Hypothetically ‘Unobserved’ confounders**	Pupillary reactivity SAH TCDB CT classification

Glasgow Coma Scale (GCS) motor score, pupillary reactivity, the Traumatic Coma Data Bank computed tomography (TCDB CT) scan classification, the presence of subarachnoid hemorrhages (SAH) and age

CT classification is based on the Marshall classification

### Control scenarios

To illustrate the usefulness and pitfalls of IV analysis and validate our simulation, six control scenarios were used (Tables 3 and 4). The seventh scenario is what we believe is closest to the truth and gives us the opportunity to test the added value of controlling for the common cause.

**Table 3:** Overview of the 7 scenarios, including six control scenarios and the seventh scenario which gives the opportunity to test the added value of controlling for the common cause

under 7 different scenarios: 1. Null scenario: no effect of treatment 2. RCT scenario: treatment randomly assigned 3. Confounder-adjusted 4. Confounder-adjusted with instrument 5. Confounder-adjusted and subject to selection bias 6. Confounder-adjusted and subject to selection bias with instrument 7. Confounder-adjusted and subject to selection bias with instrument and common cause of instrument and outcome With scenario number 7 being what we believe to be closest to the truth.

**Table 4:**
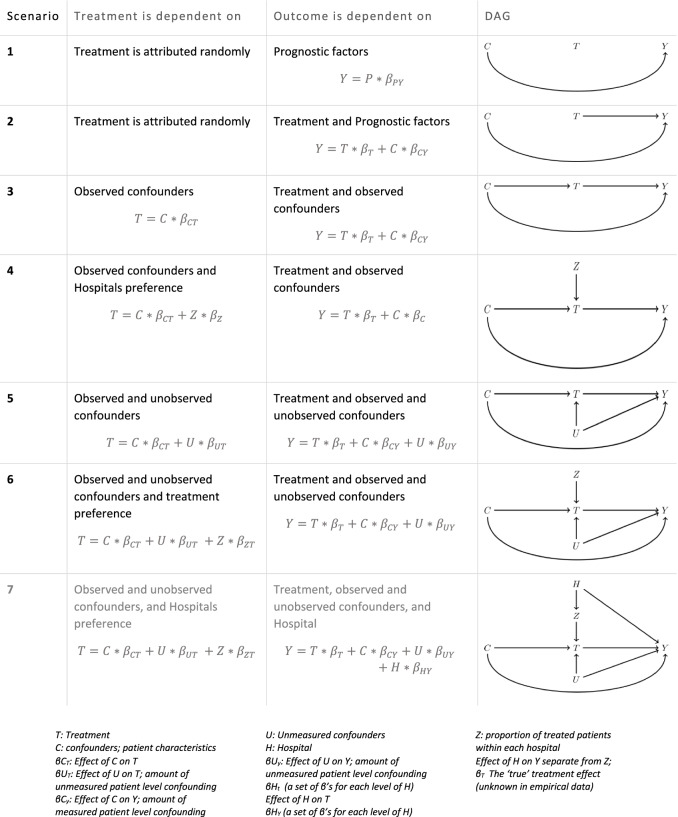
Overview of all 7 different scenarios simulated, with DAGs illustrating the assumed causal pathway

*T: Treatment*

*C: confounders; patient characteristics*

*U: Unmeasured confounders*

*H: Hospital*

*Z: proportion of treated patients within each hospital*

*βC*_*T*_*: Effect of C on T*

*βU*_*T*_*: Effect of U on T; amount of unmeasured patient level confounding*

*βC*_*y*_*: Effect of C on Y; amount of measured patient level confounding*

*βU*_*y*_*: Effect of U on Y; amount of unmeasured patient level confounding*

*βH*_*t*_ *(a set of β’s for each level of H) Effect of H on T*

*βH*_*Y*_ *(a set of β’s for each level of H) Effect of H on Y separate from Z;*

*β*_*T*_ *The ‘true’ treatment effect (unknown in empirical data)*

### IV analysis and assumptions

To test whether the first assumption *relevance* would hold, the strength of treatment preference was measured by performing a logistic regression with treatment as dependent variable and treatment preference as independent treatment variable. To assess whether the IV can be regarded as randomly assigned, the population was compared as stratified by the treatment versus stratified by the IV [20, 22].

In the adjusted IV analysis (e) the treatment preference and the hospital were the two independent variables. With the use of adjustment for hospital in the IV analysis we take into account other differences between the hospitals, such as other treatment policies that would independently affect the outcome and thus the estimated treatment effect.

### Analysis

The simulation of the data and the analyses were repeated 20,000 times. The treatment effects estimated in all analyses were expressed as Beta’s (BE) and standard errors (SE). The mean estimated treatment effects were compared to the simulated treatment effect to assess the validity of the different strategies for the analysis. To compare the different approaches in terms of precision, the standard error of the treatment effect estimates were compared between the different approaches. Further, the point estimates of 20,000 simulations were plotted for the IV and adjusted IV analysis.

All simulations and analyses were performed in R statistical software R version 3.3.0 (2016-05-03) using the following packages: rms, lme4, ordinal, and memisc [23–28].

## Results

### Patient characteristics

**Fig. 2:**
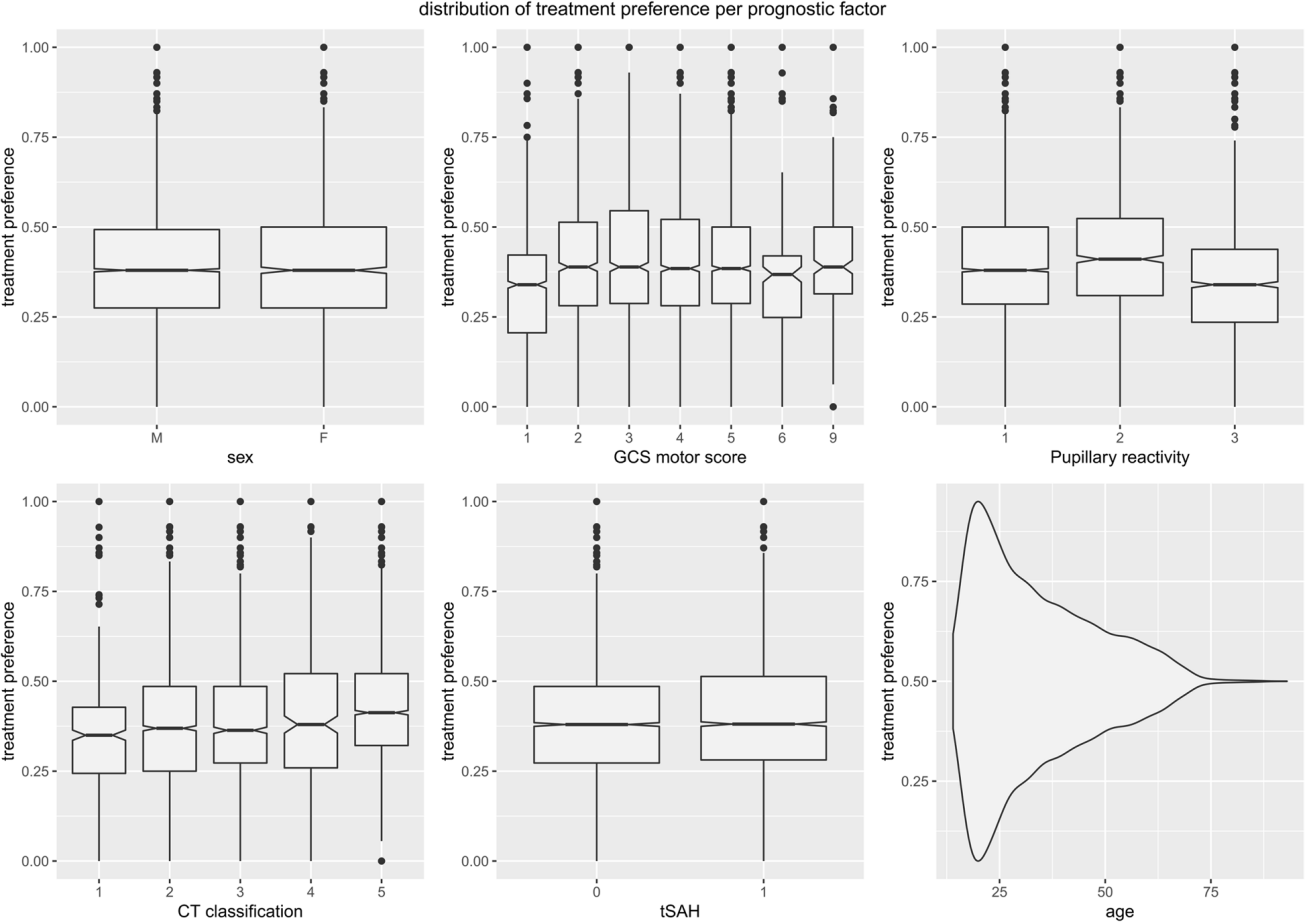
Distribution of the IV (treatment preferences) plotted per prognostic factor in the motivating example showing the distribution of the treatment preference of the hospitals attributed to each patient, per level of the prognostic factor: sex, Glasgow Coma Scale (GCS) motor score, pupillary reactivity, the Traumatic Coma Data Bank computed tomography (TCDB CT) scan classification, the presence of subarachnoid hemorrhages (SAH) and age. CT classification is based on the Marshall classification

In the original data of the IMPACT dataset 3009 (40%) of the total of 7552 patients received treatment (placement of the intracranial pressure monitor). Patients receiving an intracranial pressure monitor were generally older (median age of 34), more often male, and more often had a mass lesion (Appendix Table 5). The patient characteristics were relatively well distributed over the level of the instrument, which was percentage of patient receiving treatment in this specific hospital (Fig. 2).

### Simulation adjusted IV analysis

**Fig. 3:**
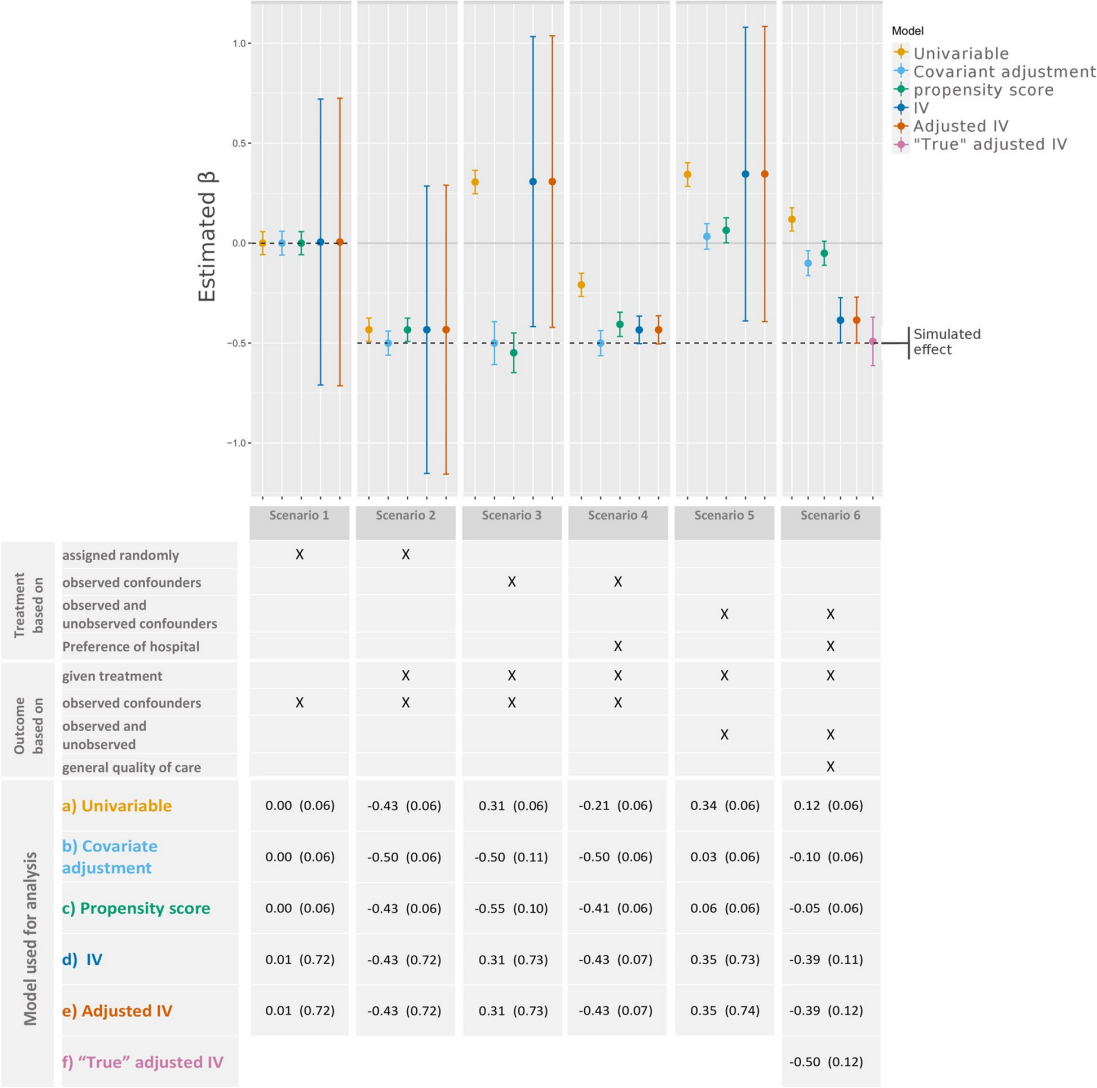
Estimated treatment effects (β) and corresponding standard errors after analysis with 5 different models in scenario 1-6: 1)Null scenario: no effect of treatment, 2) RCT scenario: treatment randomly assigned, 3) Confounder-adjusted, 4) Confounder-adjusted with instrument, 5) Confounder-adjusted and subject to selection bias, 6) Confounder-adjusted and subject to selection bias with instrument

**Fig. 4:**
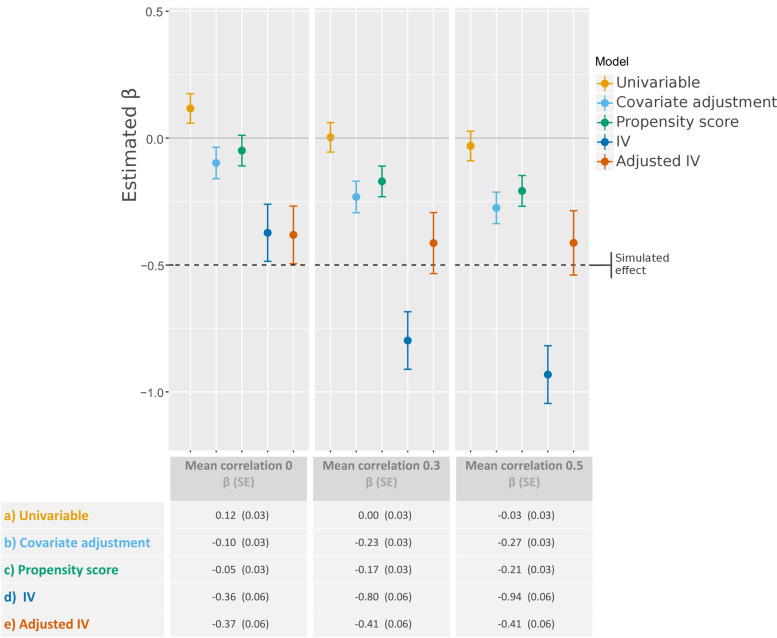
Estimated treatment effects and corresponding standard errors after analysis with no correlation between general hospital characteristics and treatment preference, and with mean correlation coefficients of 0.3 and 0.5

The simulation study showed (Fig. 3) that in the presence of unobserved confounders, the adjustment methods resulted in biased estimates (betas from 0.03 to 0.06). Using the IV analyses, betas close to the simulated treatment effect (beta = − 0.5) were estimated when there was no correlation between treatment preference and general hospital characteristics. In the scenario where treatment effect and the center effect are positively correlated (correlation of 0.3 and correlation of 0.5, Fig. 4) the conventional IV approach overestimated the treatment effect (β = − 0.80, β = − 0.93) and was significantly different from the simulated effect. Adjusted IV analysis however resulted in estimates close to the simulated effect for lower correlation (β = − 0.41) as well as for higher correlation (β = − 0.41). All IV analyses had larger SEs (0.06 vs 0.03, *n* = 15,000) than regular adjustment methods for every scenario.

Test of *relevance of the IV* (Logistic regression with treatment as dependent variable and treatment preference as independent variable) resulted in a R^2^ of 38% in the simulated data.

### Simulation for 6 different scenarios

#### Validity

Because of the presence of confounding factors in the last 4 scenarios, the unadjusted model (approach a) estimated an opposite effect of treatment in most scenarios (Fig. 3). It was only in the first 2 scenarios, which did not include confounders that the unadjusted analyses performed well.

In scenarios in which only measured confounders impacted treatment and outcome (scenario 3 and 4), covariate adjustment and propensity score matching (approach b and c) resulted in βs in the range of − 0.55 to − 0.41, with all estimates not significantly different from the simulated effect of − 0.5. However, when unmeasured confounders were present in scenarios 5 and 6, covariate adjustment and propensity score matching measured no treatment effect or an opposite effect (βs ranging from − 0.10 to 0.06).

When no confounders were included (scenario 1, 2), both the conventional (approach d) and adjusted IV (approach e) using treatment preference as an instrument did have valid results. As expected the IV methods resulted in invalid estimates in scenarios in which no treatment preference existed, but where confounders were included (β = 0.31, SE = 0.73) in scenario 3 and 5 (β = 0.35, SE = 0.73).

However, the IV analyses resulted in estimates close to the simulated effect in scenarios with both unobserved confounders and treatment preference (scenario 6). In the “true” adjustment method in scenario 6 the estimates were, as expected, exactly as simulated (β = 0.50).

#### Precision

In all scenarios, IV analyses were less reliable compared to the other adjustment methods (Figs. 3 and 4). In scenarios 1, 2, 3 and 5 no treatment preference of hospitals was simulated, therefore both IV analyses (d and e) were shown to be extremely unreliable (Fig. 3). In the scenarios in which treatment preference is simulated, the SEs of the IV analyses are smaller but remain less reliable (SE between 0.07 and 0.12) than the other adjustment methods (SE = 0.06).

**Fig. 5:**
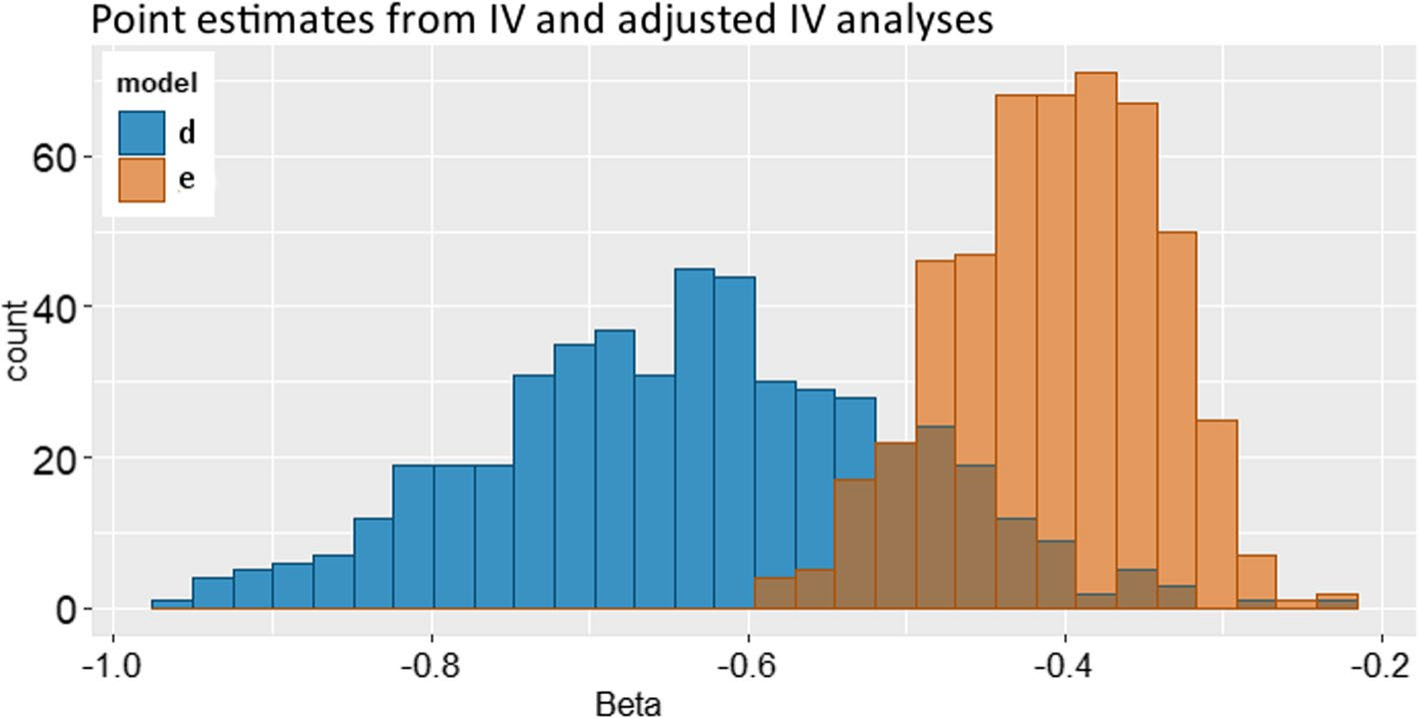
Histogram of all estimated βs in the simulations in scenario 7 of both unadjusted IV (model d) and adjusted IV (model e)

To compare IV analysis with and without adjustment we plotted the point estimates of 20,000 simulations (Fig. 5). The spread in point estimates of the adjusted IV analyses are small compared to the unadjusted IV analysis (d), which show a much larger variation. Correction for hospital in the IV in the last scenario gives more stable point estimates, comparable to regular adjustment.

## Discussion

In this study we tested the validity and precision of analyzing treatment effectiveness using IV analysis with treatment assignment per hospital as instrument compared to traditional adjustment methods, to avoid confounding by indication. Our simulation study suggests that in the presence of unobserved confounders and treatment variation between hospitals, IV analysis provides more valid treatment effect estimates compared to the regular covariate adjustment methods which correct for established prognostic factors. However, the IV analysis was considerably less reliable compared to regular adjustment. Furthermore, *adjusted IV* analysis where we adjusted for a common cause, gave valid results also when there was correlation between treatment preference and hospital characteristics, while regular IV analysis resulted in skewed estimates.

Both IV analyses estimate the effect of the treatment preference on a hospital level, and as a result, instead of the effect of the treatment we estimate the effect of the treatment preference. We note that this is a different measure and will therefore not be completely interchangeable with the effect of treatment per person. Furthermore, while over 15,000 patients were included in the analysis, and each simulated hospital was set to have 100 patients, this results in 150 hospitals being simulated. When comparing the different analyses this leads to a 100 fold difference in number of cases: *n* = 150 for IV analyses compared to *n* = 15,000 in the patient level analyses.

### Unmeasured confounders and treatment preference

We simulated 6 scenarios which build up in complexity to what we believe to be the most realistic situation. In scenario 1, an under the null scenario, treatment is attributed randomly and there is no treatment effect. Scenario 2, is what we would encounter in a randomized control trial: treatment is still attributed randomly but there is an effect of treatment. In scenario 3, we simulated the ideal observational study, where outcome and treatment are dependent on observed confounders. In scenario 4, the treatment preference is introduced, but an IV analysis is shown to be useless in this scenario, where adjustment for confounding gives the most viable results. It is only in scenario 5 when unmeasured confounders are cooperated into the analysis, that bias is introduced by using the regular adjustment methods. On the other hand the scenarios where there is no treatment preference (scenario 1,2,3 and 5) show clearly what the effect of a very weak instrument can be: in this case extremely uncertain and biased estimates from both IV analyses (d and e) which solely rely on measuring the effect of treatment preference. The estimates are, in these cases, as biased as analysis with the univariate model. The strength of the instrument can be tested and should always be considered before doing the IV analysis.

However, when unobserved confounders are included in the simulated scenarios (scenario 4 and 6), the regular adjustment methods (b and c) that only take into account measured confounders, do not sufficiently adjust for all confounders. Therefore, regular adjustment methods result in invalid treatment effect estimates in scenarios with unmeasured confounders. These scenarios, we believe are closer to the situations we face in reality, especially in the absence of evidence-based treatment recommendations. Scenario 6 includes a treatment preference per hospital as well as unmeasured confounders. It is impossible to know the degree of bias unmeasured confounders will introduce. Strong observed confounding is an indication of systematic differences between the treatment groups and thus an indication that also unobserved confounders may exist. Insight in the treatment allocation mechanisms from expert knowledge (i.e. how do doctors decide to treat or not) will provide additional information on whether unobserved confounders are expected. In our study the confounders used as unmeasured confounders just serve as an illustration to show the bias, but not to quantify the amount of bias.

In scenario 2 we see more conservative estimates for the univariate, propensity score and IV analysis. The difference between the point estimates in a scenario comparable to RCT can be ascribed to differences in the specific statistical models underlying the analyses. The regression analysis with adjustment estimates the treatment effect on patient level. This is a conditional effect estimate. The other methods estimate treatment effects on average (or the effect of treatment preference); these are marginal treatment effects, which are closer to the Null value [11, 29].

### Precision

The estimated SEs show lower precision for the IV analyses compared to regular adjustment methods in general. Point estimates also vary far more compared to regular adjustment methods in the unadjusted IV analysis (method d). The adjustment in the IV analysis seems so to solve this. IV analysis will however still require a far larger study population than in the patient level approaches to compensate for lesser statistical precision [30, 31].

### Conditions for IV analysis

Although IV analysis can result in valid treatment effect estimates, it is dependent on certain assumptions and conditions which have to be met [11]; (*relevance*) that there is an instrument and that this instrument is associated with the exposure, (*exclusion restriction*) the IV can only affect the outcome through treatment, and (*exchangeability*) the outcome and the IV cannot share a common cause. Violation of these assumptions of IV analysis can lead to different kinds of bias [32].

The need of a *relevant* IV is illustrated clearly in scenario 4 and 6 in Fig. 3. If hospitals do not base their choice to give treatment on a treatment preference, the IV analysis will estimate only noise. The extremely large SEs of the IV analysis in scenario 4 and 6, illustrate the extreme case in which the instrument has no effect at all on the outcome. The R^2^ of 0.38 shows that the treatment preference in the simulation affects the treatment, and is therefore *relevant*.

Further, the *exclusion restriction* cannot be tested, and remains an assumption based on clinical knowledge and literature [33]. When looking at the placement of intracranial pressure monitors it is possible that treatment preference could also lead to different clinically relevant choices/medication being given to the patient, which could be the true cause of the better or worse results. It is however assumed not to be the case that placing an intracranial pressure monitor will lead to different treatment choices further down the road other than those which are unavoidable after the procedure.

As for *exchangeability,* IV estimates were shown to not be biased by case-mix differences between hospitals (Table 4, Fig. 2). In our case where we suspect association between hospital performance and treatment preference, the *exchangeability* assumption is not met.

We assume that it is realistic to think there would be some (but little) correlation between treatment policies. In our case it would be imaginable that one treatment policy correlates with another treatment policy, or with certain facilities a hospital might have. The results of the IV analysis without adjustment (d) are therefore in line with what is to be expected, and show an overestimation of the treatment (preference) effect. Correcting for hospital seems to be a possible solution for analyzing data with unmeasured confounders as well as a common cause for the IV and the outcome. We see that this no longer leads to the overestimation of the treatment effect in analysis e.

In scenario 6 where a “true” adjustment was done (IV analysis but also adjustment for measured and unmeasured confounders) resulted in a point estimates exactly as the simulated treatment effect. This shows us that in the case of our simulation there was no difference in measuring the treatment vs. measuring treatment preference. It also shows that any noise measured, causing the estimate to not be exactly 0.5, is probably due to the case mix of the hospitals.

### Adjusting for hospital

In case we suspect correlation between general hospital characteristics and treatment preference we have tested the effect of adjusting for hospital, we have done this using a fixed effect model. A random effects model was not used because it would assume the covariates in the model to be independent of the exposure, since we explicitly assume they are not independent of each other, this assumption would be invalid. In these cases fixed effects models are generally advised instead of random effects models [34–36].

### Strengths and limitations

A limitation of this study is that it is based on a very specific real-life situation, we did not test a multitude of situation. The results of this study are not generalizable to other studies unless there are similar conditions. The strength of this study lies in the fact that the chosen parameters rely on real observed data. However, in the simulation study we measure an R^2^ of 0.38 for the predictability of treatment by treatment preference, while in the actual data we see a R^2^ of 0.21. Which shows that in this case our IV would not be as strong of an estimator of the real treatment since instruments which are too weak can lead to inconsistencies and bias [37, 38].

## Conclusion

In the presence of unobserved confounders IV analysis provides more valid, but less reliable treatment effect estimates compared to the regular patient-level covariate adjustment methods. The IV analysis needs a large number of patients included in a study to be able to assess treatment effects. For future research, where between center differences are used to learn about treatment effectiveness, we do recommend IV analysis if there are large differences in the policy or treatment preference to guarantee relevance of the IV. If an association between the instrument and performance of the hospital is expected, correction for hospital effects should be considered for less biased results.

### Supplementary Information

**Additional file 1.**

**Additional file 2.**

## Data Availability

The datasets generated and/or analysed during the current study are not publicly available because participants gave no consent for data sharing. A data sharing committee has been established consisting of the principal investigator (A.I.R. Maas), the lead statistical investigators (A. Marmarou, G. Murray, E. Steyerberg) and three members of the advisory board (G. Teasdale, L.F. Marshall, D. Yates), all contact information can be found at http://www.tbi-impact.org/?p=contact. Access to the IMPACT database can be granted but will be limited to use via on site facilities in the participating institutes in order to guarantee confidentiality to the original stakeholders. Relatively simple analyses, requested by other investigators, will be run by our research staff. For more complex analyses, we will provide onsite access to the database and will provide appropriate support where required. Full documentation on the content and structure of the IMPACT database will be made available. If significant time and effort is required from our research staff, costs will have to be charged to investigators requesting analyses with the IMPACT database. The R code used for simulation and analyses can be found in Additional file 2.

## Appendix

Table 5

**Table 5:** Patient characteristics in the motivating example of the International Mission for Prognosis and Analysis of Clinical Trials (IMPACT) dataset with patients grouped based on having received an intracranial pressure monitor

	Intracranial pressure monitor (*n* = 3009)	No intracranial pressure monitor (*n* = 4543)
Age (median, IQR)	34 (23-88)	27 (20-39)
Male sex	2385 (79%)	3510 (77%)
GCS motor score (median, IQR)	4 (2-5)	4 (2-5)
Pupillary reactivity		
-Both pupils reactive	1822 (61%)	3130 (69%)
-One pupil reactive	575 (19%)	580 (13%)
-No pupil reactive	612 (20%)	833 (18%)
CT classification^b^		
-Normal	42 (1%)	373 (8%)
-Diffuse II	506 (17%)	62,285 (50%)
-Diffuse III	473 (16%)	996 (22%)
-Diffuse IV	122 (4%)	180 (4%)
-Mass lesion	1866 (62%)	709 (16%)
tSAH	1511 (50%)	1904 (42%)
GOS^a^		
-Death	832 (28%)	1666 (37%)
-Persistent vegetative state	566 (19%)	977 (22%)
-Severe disability	559 (19%)	724 (16%)
-Moderate disability	161 (5%)	199 (4%)
-Good recovery	891 (30%)	977 (22%)

Table presents values after data imputation. Values are presented as n (%) unless otherwise specified

^a^ GOS: Glasgow Outcome Scale as outcome after 6 months

^b^ CT classification is based on the Marshall classification. Diffuse II refers to CT abnormalities without swelling or shift; Diffuse III refers to CT abnormalities with swelling (compressed cisterns); Diffuse IV refers to CT abnormalities with a shift

## Abbreviations

IV: Instrumental variable
RCTs: Randomized controlled trials
IMPACT: The International Mission for Prognosis and Analysis of Clinical Trials
GCS: Glasgow Coma Scale
TCDB CT: Traumatic Coma Data Bank computed tomography
*SAH*: *Subarachnoid haemorrhages*
NIHSS: National Institute of Health Stroke Scale
GOSE: Glasgow Outcome Scale
