# Supplementary material for: Instrumental variable analysis to estimate treatment effects: a simulation study showing potential benefits of conditioning on hospital
Source: BMC Med Res Methodol. 2022 Apr 25;22:121. doi: 10.1186/s12874-022-01598-6 (PMC9036707; doi:10.1186/s12874-022-01598-6)
Supplement: Supplementary file 1 — Additional file 1. [file 12874_2022_1598_MOESM1_ESM.docx]

# IMPACT database participating centers

| Fairfax Hospital, Alexandria, Virginia |
| --- |
| Erasmus MC, Rotterdam, Netherlands |
| Addenbrookes Hospital, Cambridge, United Kingdom |
| Albany Medical College, Albany, New York |
| Allegheny General Hospital, Pittsburgh, Pennsylvania |
| Barrow Neurologic Institute, Phoenix, Arizona |
| Bowman Gray School of Medicine, WinstonSalem, North Carolina |
| Branson Methodist Hospital, Kalamazoo, Michigan |
| Buffalo General Hospital, Buffalo, New York |
| Butterworth Hospital, Grand Rapids, Michigan |
| Carolina Medical Center, Charlotte, North Carolina |
| Centre Hospitalier Beaujon, Clichy, France |
| Centura St. Anthony’s Hospital Central, Denver, Colorado |
| Chaim Sheba Medical Center |
| Christ Hospital Medical Center, Oak Lawn, Illinois |
| Clinical Research Center, Sterling- Winthrop, Inc., Malvern, Pennsylvania |
| Clinique Hôpital de Rangueil, Toulouse, France |
| Fairfax Hospital, Fairfax, Virginia |
| Hadassah Hebrew University Medical Center, Jerusalem, Israel |
| Hadassah Hospital, Ein Karen, Jerusalem, Israel |
| Helsingin Yliopistollinen Keskussairaala |
| Henry Ford Hospital, Detroit, Michigan |
| Hospitais da Universidade de Coimbra, Coimbra, Portugal |
| Hospital de Hautepierre, Strasbourg, France |
| Hospital de la Citadelle, Liege, Belgium |
| Hospital Lariboisiere, Paris, France |
| Hospital Pitie-Salpetriere, Paris, France |
| Hospital Purpan, Toulouse, France |
| Hospital Trousseau, Tours, France |
| Hospital Universitario “12 De Octubre,” Madrid |
| Hospital Valle Hebron, Barcelona, Spain |
| Inselspital, Bern, Switzerland |
| J. Hollowell, Medical College of Wisconsin, Milwaukee, Wisconsin |
| Karolinska Sjukhuset, Stockholm, Sweden |
| Kennestone Hospital, Marietta, Georgia |
| Kern Medical Center, Bakersfield, California |
| Klinikum der Christian-Albrect Universitat, Kiel, Germany |
| Klinikum Grosshadern, Munich, Germany |
| Klinikum Mannheim, Mannheim, Germany |
| Legacy Emanual Hospital and Health Center, Portland, Oregon |
| M. S. Hershey Medical Center, Hershey, Pennsylvania |
| Medical College of Georgia, Augusta, Georgia |
| Medical College of Virginia, Richmond, Virginia |
| Medical Research Institute of Delaware, Newark, Delaware |
| Medical University of South Carolina, Charleston, South Carolina |
| Medizinische Einrichtungen der Heinrich-Heine-Universitat, Dusseldorf, Germany |
| Medizinische Hochschule Hannover, Hannover, Germany |
| Methodist Hospital, Indianapolis, Indiana |
| Miami Valley Hospital, Dayton, Ohio |
| Neurochirurgische Universitat, Wurzburg, Germany |
| Neurochirurgische Universitäts Klinik, Greifswald, Germany. |
| Neurokirurgisk AFD Aalborg Sygehus SYD, Aalborg, Denmark |
| Neurokirurgiska Kliniken Akademiska Sjukhuset, Uppsala, Sweden |
| Neurological Institute of Savannah, Savannah, Georgia |
| Neurological Institute, Phoenix, Arizona |
| Nevrokirurgisk Avdeling Regionsykehuset I Trondheim, Trondheim, Norway |
| Oregon Health Sciences University, Portland, Oregon |
| Orlando Regional Medical Center, Orlando, Florida |
| Ospedale Borgo Trento, Verona, Italy |
| Ospedale San Raffaele, Milan, Italy |
| Rambam Medical Center |
| Rigshospitalet, Blegdamsvej, Copenhagen, Denmark |
| Royal Victoria Hospital, Belfast, United Kingdom |
| Sacred Heart, Spokane, Washington |
| San Francisco General Hospital, San Francisco, California |
| Soroka Medical Center, Beersheba, Israe |
| Southampton General Hospital, Shirley, Southampton, United Kingdom |
| Spine Care Associates of Tyler, Tyler, Texas |
| St. Paul-Ramsey Medical Center, Rapid City, South Dakota |
| St. Vincent's Mercy Medical Center, Toledo, Ohio |
| Stanford Medical Center, Stanford, California |
| Tel Aviv Medical Center, Tel Aviv, Israel |
| Temple University, Philadelphia, Pennsylvania |
| The Liverpool Hospital, Sydney, Australia |
| The Royal London Hospital, Whitechapel, London, United Kingdom |
| the University of California at San Diego |
| the University of Texas Medical Branch |
| the University of Virginia |
| Truman Medical Center, Kansas City, Missouri |
| U.Z. Gasthuisberg, Leuvan, Belgium |
| Universitat Gesamthochschule Essen, Essen, Germany |
| Universitatsklinik Rudolf Virchow, Berlin, Germany |
| University of Antwerp |
| University of Arkansas, Little Rock, Arizona |
| University of Brescia |
| University of California at San Diego Coordinating Center, San Diego, California |
| University of Cambridge |
| University of Cincinnati, Cincinnati, Ohio |
| University of Florida, Jacksonville, Florida |
| University of Glasgow |
| University of Heidelberg |
| University of Iowa Hospital, Iowa City, Iowa |
| University of Kentucky Chandler Medical Center, Lexington, Kentucky |
| University of Louisville, Louisville, Kentucky |
| University of Manitoba, Winnipeg, Manitoba, Canada |
| University of Maryland Hospital, Baltimore, Maryland |
| University of Maryland Medical Center |
| University of Massachusetts Medical Center, Worcester, Massachusetts |
| University of Minnesota, Minneapolis, Minnesota |
| University of Nebraska Medical Center, Omaha, Nebraska |
| University of New Mexico Medical Center, Albuquerque, New Mexico |
| University of Tennessee, Memphis, Tennessee |
| University of Utah Hospital, Salt Lake City, Utah |
| Vall d'Hebron University Hospital Barcelona |
| Vancouver Hospital and Health Science Center, Vancouver BC, Canada |
| VU University Medical Center |
| Washington Hospital Center, Washington, District of Columbia |
| Wayne State University, Detroit, Michigan |
| West Virginia University Hospital, Morgantown, West Virginia |
| Westmead Hospital, Sydney, Australia |
