# Supplementary material for: Instrumental variable analysis to estimate treatment effects: a simulation study showing potential benefits of conditioning on hospital
Source: BMC Med Res Methodol. 2022 Apr 25;22:121. doi: 10.1186/s12874-022-01598-6 (PMC9036707; doi:10.1186/s12874-022-01598-6)
Supplement: Supplementary file 2 — Additional file 2. [file 12874_2022_1598_MOESM2_ESM.docx]

library(Hmisc)

library(rms)

library(foreign)

library(lme4)

library(ordinal)

library(memisc)

library(ggplot2)

library(mice)

repeats = 500

number.of.centers = 100

opt = 6

xfold = 5

everycor = 5

correlation = 0

outcome <- as.data.frame(matrix(nrow = repeats*opt, ncol = 4))

outcome[,4] <- rep(c("noconfnob", "noconf","TxX", "TxXCenter","TxXU", "TxXUCenter"),repeats)

colnames(outcome)<-c("B", "upper", "lower", "analysis")

mod0.i = mod1.i = mod2b.i = mod3.i = mod4.i = mod5.i = mod6.i = outcome

###### Data ######

impact <- read.csv('/impact.sav', to.data.frame = TRUE, use.value.labels = TRUE)

keeps <- c("patid","d_unfav","d_gos","age","d_motor",

"i_pupil","yoi","d_mort","gender", "i_ctclas", "i_tsah", "intrsurg", "centerid")

impact = impact[complete.cases(impact$intrsurg),keeps]

impact$d_motor <- as.factor(impact$d_motor)

impact$i_ctclas <- as.factor(impact$i_ctclas)

impact$i_pupil <- as.factor(impact$i_pupil)

for (j in 0:(repeats-1)){

###### multiply database

impactinf <- sample(impact, 15000, replace = TRUE)

# recode dgos

it = (as.numeric(impactinf$d_gos) - 1)

it[it == 0] = 1

impactinf$d_gos <- it

# recode to match with mortality, higher outcome is worse

impactinf$d_gos = recode(impactinf$d_gos,

1 <- 4,

2 <- 3,

3 <- 2,

4 <- 1)

dd = datadist(impactinf)

options(datadist = 'dd')

#################

# Data finished #

#################

# models on outcome and treatment

############## outcome ##############

####### Obs outcome based on observed covars #######

Obs.i <- lrm(d_gos ~ d_motor + gender + age,

data = impactinf, maxit=100, x=T, y=T, model=T)

####### Unobs outcome based on observed covars and unobs covars #######

Unobs.i <- lrm(d_gos ~ d_motor + i_pupil + i_ctclas + i_tsah + age + yoi + gender,

data = impactinf, maxit=100, x=T, y=T, model=T)

############## Treatment model ##############

####### TxObs - treatment Tx on obs covars #######

TxObs.i <- lrm(intrsurg ~ d_motor + gender + age,

data = impactinf, maxit = 100, x = T, y = T, model = T)

####### TxUnobs - Tx on obs and unobs covars #######

TxUnobs.i <- lrm(intrsurg ~ d_motor + i_pupil + i_ctclas + i_tsah + age + yoi + gender, data = impactinf, maxit = 100, x = T, y = T, model = T)

################### start simulation ###################

# random assignment of treatment, prob:0.5[constant] within all patients

TxRandom.i <- rbinom(nrow(impactinf),1,0.5)

##### assignment of treatment,based on observed covars

kans = plogis(TxObs.i$x %*% TxObs.i$coef[2:length(TxObs.i$coef)] + TxObs.i$coef[1])

TxX.i <- ifelse(mean(kans) <= kans,1,0)

##### assignment of treatment,based on observed and unobserved covars

kans = plogis(TxUnobs.i$x %*% TxUnobs.i$coef[2:length(TxUnobs.i$coef)] + TxUnobs.i$coef[1])

TxXU.i <- ifelse(mean(kans) <= kans,1,0)

##### create centers

# random distribution of patients over centers

# centers all have similar numer of patients

center <- as.numeric(sample (1:number.of.centers,nrow(impactinf), replace=T))

impactinf$center <- center

impactinf$intrsurg <- as.numeric(impactinf$intrsurg)

##### treatment is based on center, relate to tx var in pocon data: 23-61%

mintx <- 0.1

maxtx <- 0.9

centermatrix.i <- dummy(impactinf$center, levelsToKeep=c(1:number.of.centers))

txvar.i <- runif(number.of.centers,mintx,maxtx)

#### sequence, with every 5th/3rd number of the sequence,

#### then sort those, and replace within original sequence

sortseq = seq(1, length(txvar.i), everycor)

txvar.i[sortseq] <- sort(txvar.i[sortseq])

##### treatment is based on center

kans = plogis(qlogis(prop.table(table(impactinf$intrsurg))[2]) + centermatrix.i %*% qlogis(txvar.i))

TxCenter.i <- ifelse(mean(kans) <= kans,1,0)

# based on obs covars and center

kans = plogis(TxObs.i$x %*% TxObs.i$coef[2:length(TxObs.i$coef)] + TxObs.i$coef[1] + centermatrix.i %*% qlogis(txvar.i))

TxXCenter.i <- ifelse(mean(kans) <= kans,1,0)

# based on obs & unobs covars and center

kans = plogis(TxUnobs.i$x %*% TxUnobs.i$coef[2:length(TxUnobs.i$coef)] + TxUnobs.i$coef[1] + centermatrix.i %*% qlogis(txvar.i))

TxXUCenter.i <- ifelse(mean(kans) <= kans,1,0)

#####################################################

########### FILL IN TREATMENT CHOICE #################

txlist.i <- list(TxRandom.i, TxRandom.i, TxX.i, TxXCenter.i, TxXU.i, TxXUCenter.i, TxXUCenter.i)

# options outcome: y=ygos (y based on obs covars en tx), ygosUnobs (y based on obs en unobs

# covars en tx)

# options treatment allocation: TxRandom=random, Tx=TxCenter (treatment alleen afh van centrum),

# TxX (treatment only dependet on obs covars) TxXCenter (CENTER en obs) of TxXU (treatment dependent on obs and unobs)

for (i in 1:opt) {

#################################

########### TREATMENT ###########

Tx.i <- txlist.i[[i]]

#treatment preference

Txpref.i <- rep(NA, number.of.centers)

# mean treatment

for (k in 1:number.of.centers) {

Txpref.i[k] <- mean(Tx.i[impactinf$center == k])

}

#treatment preference of hospital per patient

Txprefpat.i <- centermatrix.i %*% Txpref.i

##################################################

########### OUTCOME KEUZE INVULLEN ################

# outcome based on observed x and treatment and unobserved and center effect correlated

centercoefs <- rnorm(number.of.centers,0,log(xfold)/3.92) # 0.41

# centercoefs based on treatment var, high tx --> good outcomes

# tx var is sorted, sort outcome coefs here as well to link treatment to outcome at center level

centercoefstxdependend <- sort(centercoefs, decreasing = TRUE)

correlation <- cbind(correlation, cor(centercoefstxdependend, txvar.i))

# observed or unobserved outcomes

if (i == 1) {

# outcome only based on observed x no effect of treatment, treatment assigned randomly

Lb2.1 <- Obs.i$x %*% Obs.i$coef[4:length(Obs.i$coef)] + Obs.i$coef[1]

Lb3.1 <- Obs.i$x %*% Obs.i$coef[4:length(Obs.i$coef)] + Obs.i$coef[2]

Lb4.1 <- Obs.i$x %*% Obs.i$coef[4:length(Obs.i$coef)] + Obs.i$coef[3]

A1 = (1-plogis(Lb2.1))

A12 = (1-plogis(Lb3.1) - A1)

A123 = (1-plogis(Lb4.1) - A1- A12 )

A1234 = plogis(Lb4.1)

kans = cbind(A1, A12, A123, A1234)

ygos.i <- rMultinom(kans, 1)

y.i <- ygos.i

} else if (i == 2) {

# outcome based on observed x and effect of treatment, treatment assigned randomly

Lb2.1 <- Obs.i$x %*% Obs.i$coef[4:length(Obs.i$coef)] + Obs.i$coef[1] + (Tx.i*-0.5) # 0.557 = coefficient of Tx effect from S.G.Machado paper, J Neurotrauma 1999, ARR 10% in unfav outcome.

Lb3.1 <- Obs.i$x %*% Obs.i$coef[4:length(Obs.i$coef)] + Obs.i$coef[2] + (Tx.i*-0.5)

Lb4.1 <- Obs.i$x %*% Obs.i$coef[4:length(Obs.i$coef)] + Obs.i$coef[3] + (Tx.i*-0.5)

A1 = (1-plogis(Lb2.1))

A12 = (1-plogis(Lb3.1) - A1)

A123 = (1-plogis(Lb4.1) - A1- A12 )

A1234 = plogis(Lb4.1)

kans = cbind(A1, A12, A123, A1234)

ygos.i <- rMultinom(kans, 1)

y.i <- ygos.i

} else if(i > 2 & i < 5) {

# outcome only based on observed x and treatment

Lb2.1 <- Obs.i$x %*% Obs.i$coef[4:length(Obs.i$coef)] + Obs.i$coef[1] + (Tx.i*-0.5) # 0.557 = coefficient of Tx effect from S.G.Machado paper, J Neurotrauma 1999, ARR 10% in unfav outcome.

Lb3.1 <- Obs.i$x %*% Obs.i$coef[4:length(Obs.i$coef)] + Obs.i$coef[2] + (Tx.i*-0.5)

Lb4.1 <- Obs.i$x %*% Obs.i$coef[4:length(Obs.i$coef)] + Obs.i$coef[3] + (Tx.i*-0.5)

A1 = (1-plogis(Lb2.1))

A12 = (1-plogis(Lb3.1) - A1)

A123 = (1-plogis(Lb4.1) - A1- A12 )

A1234 = plogis(Lb4.1)

kans = cbind(A1, A12, A123, A1234)

ygos.i <- rMultinom(kans, 1)

# ygos.i <- max.col(kans)

y.i <- ygos.i

}else {

# outcome based on observed x and unobserved x and treatment

Lb2.2 <- Unobs.i$x %*% Unobs.i$coef[4:length(Unobs.i$coef)] + Unobs.i$coef[1] + (Tx.i*-0.5) # 0.557 = coefficient of Tx effect from S.G.Machado paper, J Neurotrauma 1999, ARR 10% in unfav outcome.

Lb3.2 <- Unobs.i$x %*% Unobs.i$coef[4:length(Unobs.i$coef)] + Unobs.i$coef[2] + (Tx.i*-0.5)

Lb4.2 <- Unobs.i$x %*% Unobs.i$coef[4:length(Unobs.i$coef)] + Unobs.i$coef[3] + (Tx.i*-0.5)

A1 = (1-plogis(Lb2.2))

A12 = (1-plogis(Lb3.2) - A1)

A123 = (1-plogis(Lb4.2) - A1- A12 )

A1234 = plogis(Lb4.2)

kans = cbind(A1, A12, A123, A1234)

ygosUnobs.i <- rMultinom(kans, 1)

# ygosUnobs.i <- max.col(kans)

y.i <- ygosUnobs.i

}

###################################################

########## FIT MODELS ############################

##### UNADJUSTED #####

model0.i <- lrm.fit (x = Tx.i, y = y.i)

mod0.i[j*opt+i, "B"] <- model0.i$coef[length(model0.i$coef)]

mod0.i[j*opt+i, "lower"] <- confint(model0.i)[length(model0.i$coef),1]

mod0.i[j*opt+i, "upper"] <- confint(model0.i)[length(model0.i$coef),2]

##### ADJUSTED #####

# change variable names

tempx.i <- cbind(Obs.i$x, Tx.i)

colnames(tempx.i) <- sub("=", "_", colnames(tempx.i))

colnames(tempx.i) = c(colnames(tempx.i)[-ncol(tempx.i)], "tx")

model1.i <- lrm.fit (x = tempx.i, y = y.i) # adjusted

mod1.i[j*opt+i, "B"] <- model1.i$coef[length(model1.i$coef)]

mod1.i[j*opt+i, "lower"] <- confint(model1.i)[length(model1.i$coef),1]

mod1.i[j*opt+i, "upper"] <- confint(model1.i)[length(model1.i$coef),2]

##### PS ADJUSTMENT #####

PS2.i <-glm.fit(x=Obs.i$x,y=Tx.i)

model2b.i <- lrm.fit (x = cbind(PS2.i$linear.predictor, Tx.i), y = y.i) #

mod2b.i[j*opt+i, "B"] <- model2b.i$coef[length(model2b.i$coef)]

mod2b.i[j*opt+i, "lower"] <- confint(model2b.i)[length(model2b.i$coef),1]

mod2b.i[j*opt+i, "upper"] <- confint(model2b.i)[length(model2b.i$coef),2]

##### IV #####

obsix <- Obs.i$x

obsix <- factor(obsix %*% (1:ncol(obsix)))

model3.i <- lrm.fit (x = as.data.frame(Txprefpat.i), y = y.i)

mod3.i[j*opt+i, "B"] <- model3.i$coef[length(model3.i$coef)]

mod3.i[j*opt+i, "lower"] <- confint(model3.i)[length(model3.i$coef),1]

mod3.i[j*opt+i, "upper"] <- confint(model3.i)[length(model3.i$coef),2]

##### ADJUSTED IV #####

temp4.i <- as.data.frame(cbind(as.factor(impactinf$center), Txprefpat.i))

colnames(temp4.i) <- c("center", "Txpref")

model4.i <- lrm.fit (x = temp4.i, y = y.i) #

mod4.i[j*opt+i, "B"] <- model4.i$coef[length(model4.i$coef)]

mod4.i[j*opt+i, "lower"] <- confint(model4.i)[length(model4.i$coef),1]

mod4.i[j*opt+i, "upper"] <- confint(model4.i)[length(model4.i$coef),2]

if (i == 6){

##### ADJUSTED IV #####

temp5.i <- as.data.frame(cbind(as.factor(impactinf$center),Unobs.i$x, Txprefpat.i))

colnames(temp5.i) <- sub("=", "_", colnames(temp5.i))

colnames(temp5.i) = c(colnames(temp5.i)[-ncol(temp5.i)], "tTxpref")

model5.i <- lrm.fit (x = temp5.i, y = y.i) #

mod5.i[j*opt+i, "B"] <- model5.i$coef[length(model5.i$coef)]

mod5.i[j*opt+i, "lower"] <- confint(model5.i)[length(model5.i$coef),1]

mod5.i[j*opt+i, "upper"] <- confint(model5.i)[length(model5.i$coef),2]

}

}

}
